# Novel Method for the Separation of Male and Female Gametocytes of the Malaria Parasite *Plasmodium falciparum* That Enables Biological and Drug Discovery

**DOI:** 10.1128/mSphere.00671-20

**Published:** 2020-08-12

**Authors:** Melanie C. Ridgway, Kwong Sum Shea, Daniela Cihalova, Alexander G. Maier

**Affiliations:** a Research School of Biology, The Australian National University, Canberra, Australia; University of Copenhagen

**Keywords:** *Plasmodium falciparum*, malaria, gametocytes, transmission, genetic marker, sex

## Abstract

We developed a flow-cytometry-based method to separate and collect cocultured male and female Plasmodium falciparum gametocytes responsible for malaria transmission. The purity of the collected cells was estimated at >97% using flow cytometry, and sorted cells were observed by Giemsa-stained thin-smear and live-cell fluorescence microscopy. The expression of validated sex-specific markers corroborated the sorting strategy. Collected male and female gametocytes were used to confirm three novel sex-specific markers by quantitative real-time PCR that were more enriched in sorted male and female gametocyte populations than existing sex-specific markers. We also applied the method as a proof-of-principle drug screen that allows the identification of drugs that kill gametocytes in a sex-specific manner. Since the developed method allowed for the separation of male and female parasites from the same culture, we observed for the first time a difference in development time between the sexes: females developed faster than males. Hence, the ability to separate male and female gametocytes opens the door to a new field of sex-specific P. falciparum gametocyte biology to further our understanding of malaria transmission.

**IMPORTANCE** The protozoan Plasmodium falciparum causes the most severe form of human malaria. The development of sexual forms (so-called gametocytes) is crucial for disease transmission. However, knowledge of these forms is severely hampered by the paucity of sex-specific markers and the inability to extract single sex gametocytes in high purity. Moreover, the identification of compounds that specifically affect one sex is difficult due to the female bias of the gametocytes. We have developed a system that allows for the separation of male and female gametocytes from the same population. Applying our system, we show that male and female parasites mature at different rates, which might have implications for transmission. We also identified new sex-specific genes that can be used as sex markers or to unravel sex-specific functions. Our system will not only aid in the discovery of much needed gametocidal compounds, but it also represents a valuable tool for exploring malaria transmission biology.

## Abstract (precis)

The protozoan Plasmodium falciparum causes the most severe form of human malaria. The development of sexual forms (so-called gametocytes) is crucial for disease transmission. However, knowledge of these forms is severely hampered by the paucity of sex-specific markers and the inability to extract single sex gametocytes in high purity. Moreover, the identification of compounds that specifically affect one sex is difficult due to the female bias of the gametocytes. We have developed a system that allows for the separation of male and female gametocytes from the same population. Applying our system, we show that male and female parasites mature at different rates, which might have implications for transmission. We also identified new sex-specific genes that can be used as sex markers or to unravel sex-specific functions. Our system will not only aid in the discovery of much needed gametocidal compounds, but it also represents a valuable tool for exploring malaria transmission biology.

## INTRODUCTION

Transmission of malaria depends on the switch from asexual proliferation to male and female gametocytogenesis in the human host. Unlike asexual blood-stage parasites that are responsible for the symptoms of malaria, male and female gametocytes persist in the blood for weeks after symptoms cease (1, 2), waiting for mosquito ingestion to activate development to the next life cycle stage of the parasite. Eliminating gametocytes and monitoring their prevalence in countries of endemicity is crucial to eradicating malaria (3, 4).

For this reason, there has recently been a concerted effort to develop gametocyte-killing compounds. Currently, there is only one course of transmission-blocking treatment recommended by the World Health Organization: 0.25 mg/kg of primaquine (5), which poses its own risk due to the potential of severe side effects in people with glucose-6-phosphate dehydrogenase deficiency (6–8). The Medicines for Malaria Venture’s (MMV) target candidate profile includes transmission-blocking activity, preferably by killing gametocytes rather than mosquito-stage parasites (9). Once committed to gametocytogenesis, the parasite ceases rapid asexual replication. Targeting gametocytes reduces the risk of drug resistance developing since spontaneously occurring mutations that confer drug resistance would be selected over the timescale of the whole life cycle rather than being rapidly amplified in the human host prior to transmission. Most gametocyte-killing drug screens are not sex specific (10–12). These methods are limited by the natural 3- to 5-fold female bias of gametocytes (13–15), which may mask male-specific gametocyte-killing compounds. Killing one sex of gametocyte would be sufficient to sterilize the parasite and block transmission.

Of the few sex-specific gametocyte screens available, most rely on gametocyte activation markers (16, 17). These do not distinguish between activation-blocking and gametocyte-killing compounds; however, gametocyte-killing compounds are preferred since their application is considered more feasible (9). Ruecker et al. (18) combined a sex-specific activation assay with a non-sex-specific gametocyte-killing assay to infer sex-specific gametocyte-killing compounds. However, a more straightforward technique to screen gametocyte-killing compounds in a sex-specific manner is lacking.

The development of new methods has only recently enabled the investigation of male and female gametocyte populations. Tao et al. (19) predicted the sex-specific proteome *in silico* prior to the advent of sex-specific markers, in part by subtracting the proteome of non-gametocyte-producing Plasmodium falciparum strain Dd2 from that of the gametocyte-producing strain NF54.

Lasonder et al. (20) established the sex-specific proteome from two independent P. falciparum cell lines, each with a sex-specific marker (dynein heavy-chain PF3D7_1023100 for males and female marker P47, PF3D7_1346800). Both of these markers were tagged in the same cell line to determine the sex-specific transcriptome (20). Surprisingly the authors observed parasites expressing both the male and the female marker. Dynein heavy chain was later confirmed by quantitative real-time PCR (qRT-PCR) as being expressed in both male and female gametocytes (21). Another sex-specific proteome was published soon afterwards by Miao et al. (22) using alpha tubulin II expression to distinguish male and female gametocytes. Initially considered a male-specific gametocyte marker (23), it was also later observed at low levels in female gametocytes (24).

Overall, multiple strategies have contributed to our understanding of the sex-specific biology of male and female gametocytes, each more or less hinging on tagging a sex-specific gene. However, no method used markers that were expressed in one sex only.

Here, we describe an alternative fluorescence-activated cell sorting (FACS) method for the separation and collection of male and female P. falciparum gametocytes that express a female-specific green fluorescent protein (GFP) tag (25). The purity measurements of male and female populations monitored by FACS, Giemsa-stained thin smears, and live cell fluorescence microscopy are presented. The sorting strategy is also confirmed by measuring the expression of validated sex-specific markers by qRT-PCR in collected populations. The method is finally applied to validate three novel sex-specific gametocyte markers for qRT-PCR and screen for sex-specific gametocyte-killing compounds.

## RESULTS

### Method development for the enrichment of large populations of female and male gametocytes at high purity.

**FIG 1:**
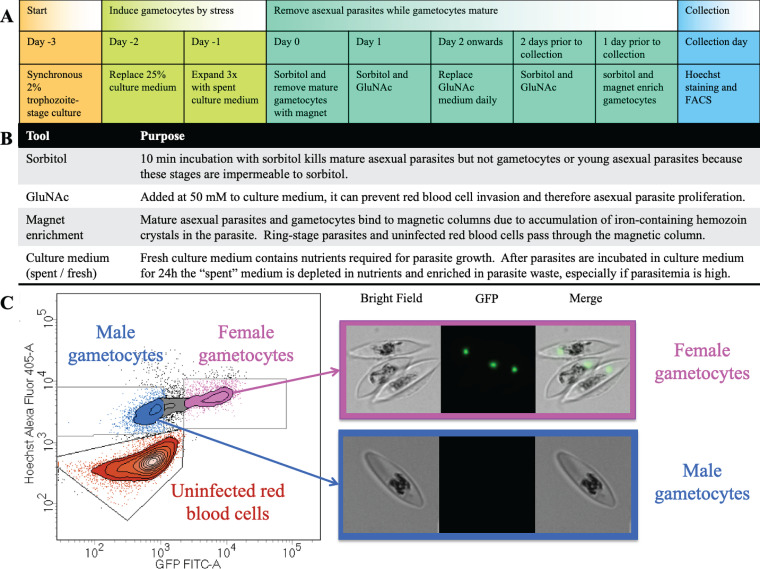
Generation and collection of male and female gametocytes. (A) Timeline of gametocyte culturing technique. (B) Explanation of terms. GluNAc, *N-*acetyl-d-glucosamine. (C) Representative FACS gating strategy of male and female gametocytes (left) based on Hoechst 33342 staining detected in the Alexa Fluor 405 channel and GFP signal detected in the FITC channel. Live collected female (top right) and male (bottom right) gametocytes show GFP fluorescence (green) imaged by DeltaVision microscopy.

In order to generate populations of highly purified male and female gametocytes, we used a cell line that expresses a GFP-tagged version of the female-specific gABCG2 protein (25). gABCG2 is located in a singular round structure in the cytoplasm of female gametocytes from stage I onward. The fluorescence signal from this marker alone allows the separation of female gametocytes from a mixed culture. However, in order to collect larger populations of viable male and female gametocytes from the same culture via FACS, we developed a protocol that enriches sexual forms before the cell sorting. Treatment of the culture with *N-*acetyl-d-glucosamine prevents the invasion of asexual merozoites into red blood cells (26). A strategically timed combination of sorbitol treatment and magnet enrichment removes the majority of uninfected red blood cells (to speed up the cell sorting) and remaining asexual stages (Fig. 1A). The DNA stain Hoechst 33342 selectively stains parasitized red blood cells; hence, females are gated as GFP positive and Hoechst positive, while males are gated as GFP-negative but Hoechst-positive cells (Fig. 1C; see also Fig. S1 in the supplemental material). For each sort, the exact position of each gate was established with reference to the scatterplot of unstained and single-color controls.

10.1128/mSphere.00671-20.1 FIG S1 Representative full gating strategy for FACS of male and female gametocytes. (Top) Dot plots of sequential gating (P1 to P3) to exclude debris and doublet cells. (Middle) Dot plot and contour plots of gating around male gametocytes (male), female gametocytes (female) and uninfected red blood cells (uRBC). (Bottom) Corresponding population statistics. SSC-A, side scatter area; FSC-A, forward scatter area; FSC-H, forward scatter height; SSC-H, side scatter height; SSC-W, side scatter width; GFP FITC-A, green fluorescent protein fluorescein isothiocyanate area (488-nm excitation/530-nm emission); Hoechst Alexa Fluor 405-A, Hoechst 33342 Alexa Fluor 405 area (405-nm excitation/421-nm emission). Download FIG S1, PDF file, 0.1 MB. Copyright © 2020 Ridgway et al. 2020 Ridgway et al. This content is distributed under the terms of the Creative Commons Attribution 4.0 International license.

**FIG 2:**
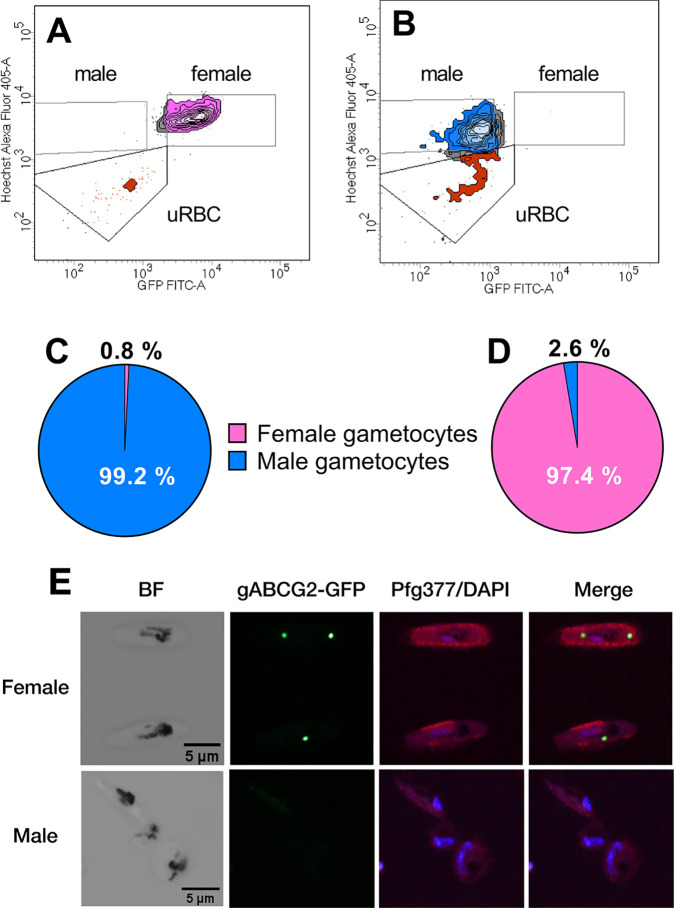
Quality controls of male/female sorting strategy. (A and B) FACS plot of re-sorted male (A) and female (B) gametocyte populations showing cells re-sorted in the female gate (pink), male gate (blue), or uninfected red blood cell gate (red) or between gates (gray). (C and D) Proportions of male population (C) or female population (D) re-sorted in the female gate (pink) or male gate (blue) averaged from 39 independent experiments. (E) Immunofluorescence microscopy of paraformaldehyde/glutaraldehyde-fixed cells in the female gate (top) and male gate (bottom) probed with anti-GFP (green, first column) and anti-Pfg377 (red, second column) antisera. The nuclei were visualized with DAPI (violet). Scale bar, 5 mm. Note that the upper female gametocyte contains two gABCG2-positive structures, which is occasionally observed (25).

The success of this approach was evaluated by visually inspecting the sorted populations by microscopy (absence or presence of GFP, morphology, and distribution of the hemozoin crystals within the cell). In addition, the purity of the sorted cells was assessed by subjecting the resulting populations to another round of cell sorting while maintaining exactly the same gating strategy. Of the cells regated as gametocytes in the male population, 99% were regated as male gametocytes (Fig. 2A and C). Upon re-sorting the female population, 97% of gametocytes were regated as females (Fig. 2B and D), indicating the purity and reproducibility of this approach. Using this culture method and gating strategy, between 2 and 12 million gametocytes of each sex were isolated from 1.2 liters of culture, representing a yield of 1.7 to 10 million gametocytes of each sex per liter of culture. Higher yields could be achieved with a less stringent gating strategy, although this would be at the expense of sample purity.

To provide further evidence for the purity of the resulting samples, we stained the separated populations with anti-Pfg377 antibodies. Pfg377 is predominantly (but not exclusively) expressed in female gametocytes, where it is located in osmiophilic bodies (27). The cells of the gABCG2-GFP-positive population showed an intense labeling pattern that is mainly observed in a punctate pattern near the cellular perimeter, consistent with the distribution of osmiophilic bodies in female gametocytes (Fig. 2E). The fluorescent signal obtained from this antibody was much weaker and more diffuse in the cells of the male population, which is consistent with the reduced Pfg377 expression in male parasites.

Together, these data show that the method allowed the highly specific separation of male and female gametocytes.

### Male gametocytes are developmentally delayed compared to female gametocytes.

**FIG 3:**
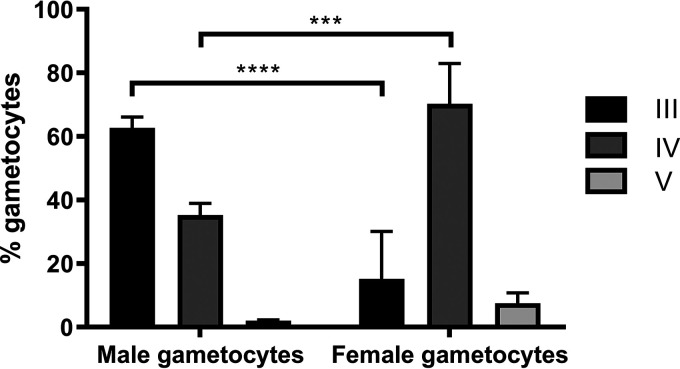
Gametocyte stage in sorted male and female populations at day 8 after commitment. After FACS, the male and female gametocyte stages were counted based on a Giemsa-stained thin smear. Means and standard deviations from three biological repeats with results from two-way ANOVA with multiple comparisons are presented. ****, *P* < 0.0001; ***, *P* < 0.001.

While sorting populations at day 8 after commitment, we observed that the vast majority of female gametocytes displayed morphological characteristics of stage IV gametocytes (elongated biconvex body with pointed extremities), as expected. However, the male gametocytes showed a delay in development and the majority of cells in this population were at stage III (as indicated by their less-elongated planoconvex or D-shaped body). In order to quantify this effect, we analyzed three independent experiments and determined microscopically the developmental stage of the cells by determining the developmental stage of 300 gametocytes in each population (Fig. 3). This confirmed that at day 8 after commitment the majority of female gametocytes were at stage IV, but the male gametocytes had developed predominantly to stage III. This effect was reproducible and statistically significant (*P* < 0.001 in two-way analysis of variance [ANOVA]) for 3D7 strain gABCG2-GFP gametocytes cultured *in vitro*.

### Discovery and evaluation of new sex-specific markers.

**FIG 4:**
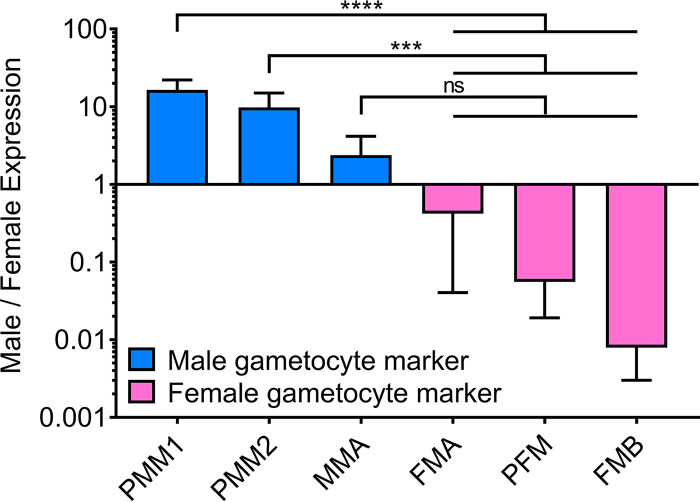
Male/female expression of male markers (blue) or female markers (pink) relative to reference gene PF3D7_0317300 measured by qRT-PCR of male and female gametocyte populations collected by FACS. The relative target gene expression is calculated as follows from four technical repeats, with *E* being the real-time PCR efficiency and CP being the crossing point, as initially proposed by Pfaffl (49): ratio = (*E*_target_^ΔCPtarget(female-male)^)/(*E*_ref_^ΔCPref(female-male)^). PMM1 and PMM2, putative male markers 1 and 2 (*Plasmodium* exported protein [PHISTa] PF3D7_1477700 and PF3D7_1438800, respectively); MMA, male marker A (P230p, 6 cystein protein PF3D7_0208900); FMA, female marker A (gametocyte ABC transporter G family member 2 PF3D7_1426500); PFM, putative female marker (PF3D7_1447600); FMB, female marker B (P25, ookinete surface protein PF3D7_1031000). Means and standard deviations from four technical repeats with results from two-way ANOVA with multiple comparisons are presented. ****, *P* < 0.0001; ***, *P* < 0.001; ns, not significant.

Despite the fact that there are significant morphological and functional differences between male and female gametocytes, there are very few molecular markers described that are characteristic of one particular sex. As a reference and to evaluate the sorting method, we measured the expression of known sex-specific markers in populations of separated male and female gametocytes on day 8 after commitment by qRT-PCR of male marker P230p (PF3D7_0208900 [male marker A]) and of female markers gABCG2 (PF3D7_1426500 [female marker A]) and P25 (PF3D7_1031000 [female marker B]) (Fig. 4). In addition, based on expression profiles in PlasmoDB (20, 28), we selected a putative female marker (PF3D7_1447600 [PFM]) and two putative male markers (PF3D7_1477700 [PMM1] and PF3D7_1438800 [PMM2]). We found that the expression of the known male marker P230p in the isolated male population is only double that of the female population. However, expression of the putative male markers 1 and 2 were approximately 16- and 10-fold higher in the male population, respectively, making them good male markers for future studies. The female marker P25 had the highest expression in female populations (∼127× compared to males); however, there was also a significant signal in the males, consistent with Lasonder et al. (20). In contrast, the female marker gABCG2 and the putative female marker only showed background expression in the male population. The relative expression was ∼2× higher in females for gABCG2 and ∼18× higher for the novel putative female marker.

This approach allowed us to confidently identify novel sex-specific molecular markers that can be used for surveillance and research purposes.

### Assay reveals target window of sex-specific drug action of Malaria Box compounds.

**FIG 5:**
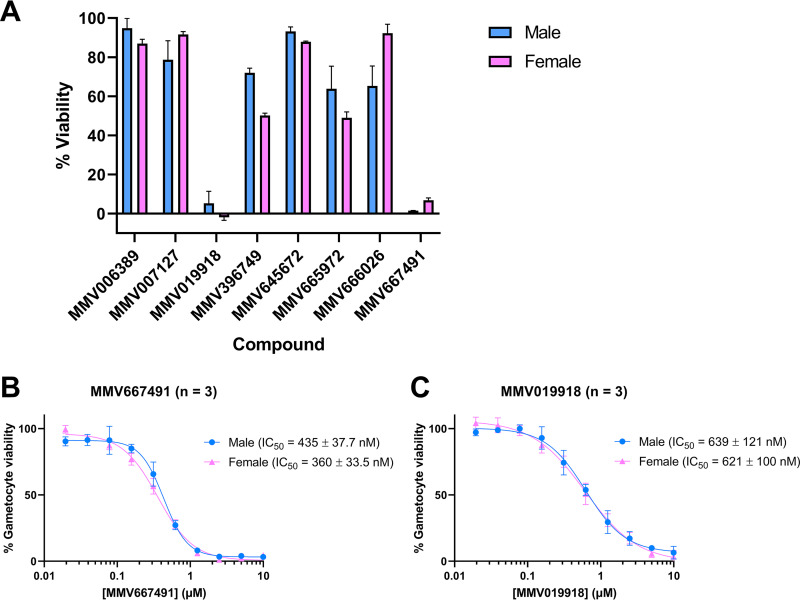
Screen of sex-specific gametocyte-killing compounds. (A) Gametocytes were exposed to 10 μM concentrations of each compound for 72 h from day 6 postcommitment (stages III and IV), after which the sex-specific viability was measured by flow cytometry. The results are normalized to the effect of the solvent control, 0.1% (vol/vol) DMSO, and known antimalarial primaquine at 100 μM. The means and standard errors of the mean (SEM) for male (blue) and female (pink) viabilities from two technical replicates are presented. (B and C) Dose-response curve (mean ± the SEM) of male (blue) and female (pink) gametocytes to MMV667491 (B) and MMV019918 (C) measured in three independent biological replicates in the same way. IC_50_, half maximal gametocyte-killing concentration.

In order to explore the suitability of the method to identify sex-specific gametocyte-killing drugs, we selected eight compounds from the MMV Malaria Box, which were previously shown to prevent the activation of male gametes but had very little effect on female gametes (18). We conducted a screen by exposing a committed culture from day 6 after commitment for 72 h at 10 μM. The viability of male and female gametocytes was assessed using a highly sensitive viability stain, MitoTracker Deep Red, which only stains mitochondria with a membrane potential, i.e., cells with an active electron transport chain. The parasite viability was measured relative to that of parasites exposed to the dimethyl sulfoxide (DMSO) control (no effect) and primaquine control (100% lethal effect) (Fig. 5A). Although primaquine is activated *in vivo*, we used >5-fold the 50% inhibitory concentration (IC_50_) previously described for the nonactivated form against gametocytes (11, 29). We also compared the activity of 100 μM primaquine against 200 μM artemisinin and found comparable killing effects (see Fig. S2). Six of the compounds did not result in a reduction of the gametocyte viability of >50%. Two compounds (MMV019918 and MMV667491), however, killed >95% of the gametocytes. We performed a dose-response growth assay on all compounds that killed >80% of gametocytes at 10 μM, which revealed that neither of the two compounds is male specific at this development stage. Together with previous findings that these compounds inhibit male gamete activation (18), these results showed that the target of these compounds affecting predominantly male gametes must be specific to late stage male gametocytes/activating gametes and is absent in early- to mid-stage gametocytes.

10.1128/mSphere.00671-20.2 FIG S2 Comparison of the effect of 100 μM primaquine and 200 μM artemisinin on the viability of gametocytes. Using the gametocyte viability assay no significant difference between the two drugs is observed. To test the sex-specific effect of drugs, the data shown in Fig. 5 was normalized by setting the background readings (residual MitoTracker Deep Red signal after exposure to primaquine) to 0% and the readings obtained from cultures grown in 0.1% (vol/vol) DMSO to 100%. Means and standard deviations from three biological repeats with results from two-way ANOVA with multiple comparisons are presented. ***, *P* < 0.001; ns, not significant. Download FIG S2, EPS file, 0.8 MB. Copyright © 2020 Ridgway et al. 2020 Ridgway et al. This content is distributed under the terms of the Creative Commons Attribution 4.0 International license.

## DISCUSSION

We performed an in-principle drug assay to investigate whether previously described sex-specific compounds affecting male gamete activation also have a sex-specific effect on early gametocytes. Of the eight compounds tested, only two killed >80% of gametocytes at 10 μM. When we determined the sex-specific IC_50_ for each of these two compounds, the effect did not seem to be sex specific. Hence, the assay revealed that (i) the targets for the two compounds that killed male and female gametocytes and block male gamete activation is essential for gametocytes maturation and male gamete activation and (ii) for the six compounds that had no significant effect on gametocytes, their target is only essential for male gamete activation.

Hence, the assay revealed that the targets for the compounds that interfere specifically with male gamete activation are essential in males specifically for gamete activation but not for the sex-specific development of gametocytes. Applying our method to an in-principle drug screen showed that the collected male and female gametocytes are amenable to the identification of sex-specific gametocyte-killing drugs and their biological targets.

Killing one sex of gametocytes is sufficient to sterilize the parasite and block transmission; hence, such a strategy might be highly specific with fewer side effects and might also curb the spread of resistance against this compound. For example, this protocol could be adjusted for sorting into drug assay plates for high-throughput screens on mature gametocytes or cultures could be drug treated prior to sorting to measure sex-specific effects.

An effective drug development strategy against the malaria parasite P. falciparum involves the rational design of compounds targeted to essential, parasite-specific functions. This requires a detailed understanding of the molecular traits of the different life cycle stages. In particular, the transition from human host to the mosquito and the corresponding sexual development represents a vulnerable stage for the parasite. The sex-specific transcriptome and proteome of gametocytes is now established (19–22, 30), but the metabolomics evidence is limited to combined male and female gametocytes (31–34). The method presented here enables us to separate populations of male and female gametocytes at high purity from the same culture and therefore will allow us to close this gap.

With some modifications, the presented method will be even more versatile. The use of only a female-specific marker (and consequently the relatively large safety margin that is used to distinguish between the male and female populations in the gating strategy) prevents the presented protocol from being used to directly determine the sex ratio in the population. The protocol was designed with purity in mind, at the expense of gametocyte yield. However, tagging one of the identified male-specific markers with another fluorophore in the GFP-labeled gABCG2 cell line used here should overcome these limitations.

The ability to separate male and female gametocytes has also enriched the available repertoire of sex-specific molecular markers: RNA extracted from collected male and female gametocyte populations validated three novel sex-specific markers by qRT-PCR. Currently, gametocyte carriage is measured with P25, a female-specific transcript. Initially P25 was routinely used as a generic gametocyte marker; however, it was later found to be highly enriched in female gametocytes (20, 35). Male markers were subsequently included in the sex-specific gametocyte measurements (36–40).

In particular, two of the newly identified sex-specific markers in this study hold great promise: transcripts of the putative male marker 1 (PMM1 PF3D7_1477700) are >10-fold more abundant than the currently used male marker P230p, and the ratio between male and female transcripts is 144 (>3-fold higher than P230p; see Table S2) (20). The transcripts of the putative female marker (PFM PF3D7_1447600) are abundant (although significantly less than the transcripts for the female marker p25). At the same time, the ratio between female and male gametocytes is 1.6-fold higher than for p25. These values indicate very good sensitivity and improved specificity in comparison to the currently used markers.

10.1128/mSphere.00671-20.4 TABLE S2 Expression levels of known and putative sex-specific markers as determined in previous transcriptomic studies (20, 28). Values are expressed as fragments per kilobase of transcript per million mapped reads (FPKM) (except for the right column that displays the sex-specific ratio of each gene). Download Table S2, DOCX file, 0.01 MB. Copyright © 2020 Ridgway et al. 2020 Ridgway et al. This content is distributed under the terms of the Creative Commons Attribution 4.0 International license.

Ultimately, a reliable means for measuring male and female gametocyte density and sex ratio may predict the transmissibility of the parasite, currently measured by expensive and labor-intensive membrane feeding assays (15). These sex-specific markers could be used to monitor gametocyte sex ratios in the field or in the characterization of genetically modified cell lines generated in the laboratory. To our knowledge, none of these novel sex-specific genes have been characterized beyond being identified in high-throughput screens. The sex-specific role of these genes warrants further investigation.

Our study also exemplified the potential of this method to reveal important biological concepts: the unexpected finding that female and male gametocytes develop at different rates highlights the advantage of using the same cell line to isolate both populations. We cannot explain this phenomenon as an artifact of the method itself. Following P. falciparum infection of human volunteers by mosquito bite in a recent clinical trial (41), female gametocytes were detected earlier than male gametocytes by qRT-PCR (mean of 13.9 days compared to 17.3 days after the first detection of asexual blood stages). Given that only mature stage V gametocytes appear in circulation *in vivo*, this is consistent with the developmental delay between male and female gametocytes observed in the present study. Biologically, a difference in development times makes self-fertilization in a high-transmission scenario less likely and hence increases recombination. Sufficient recombination rates are important to provide genetic flexibility for the parasite to respond to environmental changes. In a low-transmission situation the parasite could still be transmitted and self-fertilize since, on average, gametocytes would spend more time within the host before being taken up by mosquitoes. During this time, the male gametocytes are able to catch up in their development and become mature enough for gamete activation and fertilization. This scenario prioritizes successful transmission (and therefore survival) of the parasites over the long-term gain of genetic variability. This hypothesis concurs with the findings that the rate of recombination is higher in high-transmission areas (42, 43). Hence, different rates by which the sexes become transmission competent allow a balance between sufficient recombination rates and ensure the transmission and survival of the parasite.

Ultimately, a better understanding of the sex-specific biology of gametocytes gleamed from the analysis of gametocytes separated using this method could lead to better malaria transmission-blocking strategies in the field and further our understanding of the sexual development of P. falciparum parasites.

## MATERIALS AND METHODS

### Cell lines used in this study.

P. falciparum 3D7 strain parasites expressing a GFP-tagged gametocyte ATP-binding cassette transporter family member 2 (gABCG2) protein (PlasmoDB accession no. PF3D7_1426500) previously described by Tran et al. (25) was used throughout. FACS calibration by GFP-negative cells was performed with the parental P. falciparum 3D7 wild-type parasites.

### Culture of male and female gametocytes.

Asexual parasites were cultured as described by Maier and Rug (44) and induced to form gametocytes as described by Fivelman et al. (45) with modifications to reduce asexual parasite proliferation. Briefly, sorbitol synchronized trophozoite-stage parasites at 2% parasitemia were incubated overnight and “stressed” by replacing only a quarter of the culture medium. Two-thirds of the culture medium and red blood cells were replenished the next day. On the following day, termed day 0 of gametocytogenesis, a high parasitemia ring-stage culture was treated with sorbitol to remove asynchronous trophozoites as described by Lambros and Vanderberg (46).

To remove the remaining mature asexual parasites and mature gametocytes, the culture was then twice passed through a MACS CS column placed in the magnetic field of a SuperMACS II separator according to the manufacturer’s instructions. From day 1 onward, the culture medium was replaced daily and supplemented with 50 mM *N-*acetyl-d-glucosamine to prevent asexual parasite proliferation (26). To collect gametocytes on day 9, the culture was treated with sorbitol on day 7 and 8 to remove asexual parasites. On day 8, the gametocytes were enriched using a MACS CS column in the magnetic field of a SuperMACS II separator according to the manufacturer’s instructions, this time collecting the magnetic fraction (containing the gametocytes) and discarding the flowthrough (containing uninfected red blood cells). Note that magnetic enrichment of gametocytes improves the gametocyte sorting efficiency but is not necessary for small gametocyte collections since uninfected red blood cells can also be excluded by FACS (see below). Gametocytes were eluted in excess culture medium and incubated overnight in normal culturing conditions.

### FACS collection of male and female gametocytes.

Gametocytes were incubated with 50 μg/ml Hoechst 33342 (Invitrogen) in phosphate-buffered saline (PBS) for 15 min and then rinsed twice in PBS using 2,000 × *g*, 1-min spins and resuspended in PBS for FACS. A subset of gametocytes were resuspended in PBS but not stained for a GFP-only control. Asexual 3D7 wild-type parasites were resuspended in PBS for the unstained control and stained with Hoechst 33342 as described above to produce a Hoechst-only control. Sorts were performed on a FACSAria I calibrated prior to each sort with the unstained and single-color controls. After initially confirming that gametocyte Hoechst staining intensity is similar to that of ring-stage asexual parasites, asexual cultures were subsequently used for unstained and Hoechst-only controls for convenience (rather than committing a wild-type culture to gametocytogenesis).

The gating strategy established using FACSDiva software is illustrated in Fig. S1 in the supplemental material. Briefly, three gates were drawn to isolate whole single cells based on forward and side scatter. Of these cells, all gametocytes were identified based on the presence of Hoechst staining, while females displayed an additional GFP signal from the female-specific gABCG2-GFP. Male and female gates were conservatively drawn to collect the purest samples possible. In particular, Hoechst-positive cells with low GFP intensity were excluded from both male and female gates. FACS was performed at 37°C in PBS with collection in culture medium diluted up to 1:5 by PBS droplets during collection. Collected cells were centrifuged at 1,800 × *g* for 10 min and used immediately for experiments.

### Microscopy of male and female gametocytes.

Live cells in PBS were observed at 100× magnification with immersion oil on a restorative widefield deconvolution microscope (DeltaVision Elite; GE Healthcare Australia) detecting GFP (488-nm excitation/498- to 598-nm emission) and Hoechst 33342 (405-nm excitation/410- to 498-nm emission). Images were deconvolved using the softWoRx acquisition software (ve5.0) and were processed with the Fiji module in ImageJ 2.0 software (National Institutes of Health).

For the labeling with anti-Pfg377 antibodies, an indirect immunofluorescence assay was performed. Sorted parasites were allowed to adhere to concanavalin A-coated microscopy slides for 15 min before being fixed in 2% (vol/vol) paraformaldehyde–0.008% (vol/vol) glutaraldehyde for 20 min. The cells were washed in PBS and then permeabilized in 0.1% (wt/vol) Triton X-100 (Sigma) for 10 min before being incubated in rabbit anti-GFP (1:1,000; a gift from Mike Ryan, Monash University) and mouse anti-Pfg377 antibodies (1:500; a gift from Pietro Alano, Istituto Superiore di Sanita Roma) in 3% (wt/vol) bovine serum albumin/PBS for 2 h. The cells were washed in PBS before being incubated with secondary antibodies conjugated to the fluorophores Alexa Fluor 488 (anti-rabbit [green]; Thermo Fisher) and Alexa Fluor 594 (anti-mouse [red]; Thermo Fisher) (diluted 1:2,000 in 3% [wt/vol] bovine serum albumin/PBS) for 1 h. Mounting took place in Vectashield containing DAPI (4′,6′-diamidino-2-phenylindole; Vector Laboratories) and examined with a restorative widefield deconvolution microscope (DeltaVision Elite; GE Healthcare Australia) using a 100× objective (1.4 NA). Images shown are maximum projection images of whole-cell z stacks (0.2-μm intervals) using softWORx acquisition software (v5.0) and processed with ImageJ 1.43u software (National Institutes of Health). Acquisition settings were kept the same for all samples, and color, brightness, and contrast were uniformly modified for clarity.

### qRT-PCR of male and female gametocytes.

RNA was extracted from collected male and female gametocytes and saponin-isolated trophozoites (47) by using an RNeasy minikit (Qiagen). The remaining genomic DNA was removed, and cDNA was synthesized using a QuantiTect reverse transcription kit (Qiagen) and stored at –20°C.

A reference gene and markers for male and female gametocytes were selected based on RNA-seq profiles on PlasmoDB (20, 28). Of the selected markers, female marker A (PF3D7_1426500, gABCG2) (25), female marker B (PF3D7_1031000, ookinete surface protein P25) (35), and male marker A (PF3D7_0208900, 6 cysteine protein) (48) were previously described sex-specific markers. Putative female marker (PF3D7_1447600), putative male marker 1 (PF3D7_1477700, *Plasmodium* exported protein PHISTa), and putative male marker 2 (PF3D7_1438800) are novel markers. The reference marker (PF3D7_0317300) was chosen due to its equal transcription levels across both the sexual and asexual red blood cell life cycle stages of P. falciparum (20, 28). Primer pairs were designed by the Integrated DNA Technologies (IDT) Primer Quest Tool using the 3D7 strain gene sequences from PlasmoDB (see Table S1 in the supplemental material). Primers from IDT were resuspended in 10 mM Tris–1 mM EDTA buffer and stored at –20°C.

10.1128/mSphere.00671-20.3 TABLE S1 Forward and reverse qRT-PCR primers used in this study for each targeted parasite stage and gene marker. FMA, female marker A; FMB, female marker B; PFM, putative female marker; MMA, male marker A; PMM1, putative male marker 1; PMM2, putative male marker 2. Download Table S1, DOCX file, 0.01 MB. Copyright © 2020 Ridgway et al. 2020 Ridgway et al. This content is distributed under the terms of the Creative Commons Attribution 4.0 International license.

qRT-PCR was performed with a Light Cycler 480 (LC480) SYBR green I Master (Roche) according to the manufacturer’s instructions with 10-μl reactions in a 384-well plate. Thermocycling conditions were as follows: 10 min, 95°C preincubation; 45 cycles of 15-s denaturation at 95°C, 15-s annealing at 50°C, and 20-s elongation at 72°C; followed by a melting curve established by denaturing at 95°C for 30 s, annealing at 60°C for 30 s, and then slowly denaturing by increasing the temperature to 95°C at 0.11°C/s. Melting curves were observed using LC480 software. The exported text file from LC480 was converted using LC480 converter (http://www.hartfaalcentrum.nl/index.php?main=files&sub=0) and quantitation cycles (*C_q_*), and PCR efficiencies were determined in LinReg (http://www.hartfaalcentrum.nl/index.php?main=files&fileName=LinRegPCR.zip&description=LinRegPCR:%20qPCR%20data%20analysis&sub=LinRegPCR). The relative quantification of transcripts was expressed as previously described (49). Two-way ANOVA was performed in GraphPad Prism 8.

### Sex-specific gametocyte viability assay.

To determine the sex-specific effect of MMV Malaria Box compounds on gametocytes, we selected eight compounds that were previously shown to specifically affect the activation of male gametes (18).

Magnet-enriched gABCG2-GFP gametocytes were exposed to 10 μM concentrations of each compound, 100 μM primaquine (0% viable control), or 0.1% (vol/vol) DMSO (100% viable control) for 72 h from day 6 postcommitment in culture medium with 50 mM *N*-acetyl-d-glucosamine in a 96-well plate at 37°C in hypoxic conditions (1% O_2_, 5% CO_2_, 94% N_2_). The half-maximal killing concentration (IC_50_) of a subset of compounds was determined by exposing gametocytes to a gradient of drug concentrations under the same conditions.

Parasites were stained with 5 μg/ml Hoechst 33342 and 500 nM MitoTracker Deep Red FM in PBS with 10 mM d-glucose for 30 min at 37°C in hypoxic conditions (1% O_2_, 5% CO_2_, 94% N_2_). Stained cells were rinsed twice in PBS with 1,000 × *g*, 5-min spins and resuspended in PBS with 10 mM d-glucose. Single-color controls for flow cytometry consisted of asexual 3D7 wild-type culture stained with 500 nM MitoTracker Deep Red FM or 5 μg/ml Hoechst 33342 for 30 min in PBS supplemented with 10 mM d-glucose and rinsed twice in PBS with 1,000 × *g*, 1-min spins and then resuspended in PBS with 10 mM d-glucose. Unstained 3D7 gABCG2-GFP gametocytes were resuspended in PBS with 10 mM d-glucose. Unstained asexual 3D7 wild-type parasites in PBS with 10 mM d-glucose were used as the unstained control. Samples were analyzed on a LSR II flow cytometer (BD Biosciences) detecting Hoechst 33342 in the Pacific Blue channel (405-nm excitation/461-nm emission), MitoTracker Deep Red in the APC-Cy7 channel (644-nm excitation/665-nm emission), and GFP in the fluorescein isothiocyanate (FITC) channel (488-nm excitation/509-nm emission). A total of 200,000 events were recorded in each single-color control, and 100,000 events were recorded in each sample.

The gating strategy to exclude debris, select single cells, exclude uninfected red blood cells and asexual parasites, and identify male and female gametocytes is equivalent to that shown in Fig. S1 in the supplemental material. In addition, the number of MitoTracker Deep Red FM positive events detected on the APC-Cy7 channel was recorded for each of the gametocyte populations. Data were analyzed in FlowJo, and graphs were prepared in GraphPad Prism 8. Cell viability is expressed as a percentage of the number of MitoTracker Deep Red FM positive events in samples treated with 0.1% (vol/vol) DMSO (100% viable) and in 100 μM primaquine-treated samples (0% viable).

## References

[1] Smalley ME, Sinden RE. 1977. *Plasmodium falciparum* gametocytes: their longevity and infectivity. Parasitology 74:1–8. doi:10.1017/s0031182000047478. 320542

[2] Gebru T, Lalremruata A, Kremsner PG, Mordmüller B, Held J. 2017. Life-span of *in vitro* differentiated *Plasmodium falciparum* gametocytes. Malar J 16:330. doi:10.1186/s12936-017-1986-6. 28800735 PMC5553604

[3] Alonso PL, Brown G, Arévalo-Herrera M, Binka F, Chitnis C, Collins F, Doumbo OK, Greenwood B, Hall BF, Levine MM, Mendis K, Newman RD, Plowe CV, Rodríguez MH, Sinden R, Slutsker L, Tanner M. 2011. A research agenda to underpin malaria eradication. PLoS Med 8:e1000406. doi:10.1371/journal.pmed.1000406. 21311579 PMC3026687

[4] Liu J, Modrek S, Gosling RD, Feachem RGA. 2013. Malaria eradication: is it possible? Is it worth it? Should we do it? Lancet Glob Health 1:e2–e3. doi:10.1016/S2214-109X(13)70002-0. 25103582

[5] World Health Organization. 2018. World malaria report—2018. World Health Organization, Geneva, Switzerland.

[6] Hockwald RS, Arnold J, Clayman CB, Alving AS. 1952. Toxicity of primaquine in negroes. JAMA 149:1568–1570. doi:10.1001/jama.1952.72930340027010c. 14945981

[7] Dern RJ, Beutler E, Alving AS. 1954. The hemolytic effect of primaquine. II. The natural course of the hemolytic anemia and the mechanism of its self-limited character. J Lab Clin Med 44:171–176. 13184224

[8] Recht J, Ashley EA, White NJ. 2018. Use of primaquine and glucose-6-phosphate dehydrogenase deficiency testing: divergent policies and practices in malaria endemic countries. PLoS Negl Trop Dis 12:e0006230. doi:10.1371/journal.pntd.0006230. 29672516 PMC5908060

[9] Burrows JN, Duparc S, Gutteridge WE, Hooft van Huijsduijnen R, Kaszubska W, Macintyre F, Mazzuri S, Möhrle JJ, Wells TNC. 2017. New developments in anti-malarial target candidate and product profiles. Malar J 16:26. doi:10.1186/s12936-016-1675-x. 28086874 PMC5237200

[10] D’Alessandro S, Silvestrini F, Dechering K, Corbett Y, Parapini S, Timmerman M, Galastri L, Basilico N, Sauerwein R, Alano P, Taramelli D. 2013. A *Plasmodium falciparum* screening assay for anti-gametocyte drugs based on parasite lactate dehydrogenase detection. J Antimicrob Chemother 68:2048–2058. doi:10.1093/jac/dkt165. 23645588

[11] Wang Z, Liu M, Liang X, Siriwat S, Li X, Chen X, Parker DM, Miao J, Cui L. 2014. A flow cytometry-based quantitative drug sensitivity assay for all *Plasmodium falciparum* gametocyte stages. PLoS One 9:e93825. doi:10.1371/journal.pone.0093825. 24736563 PMC3988044

[12] D’Alessandro S, Camarda G, Corbett Y, Siciliano G, Parapini S, Cevenini L, Michelini E, Roda A, Leroy D, Taramelli D, Alano P. 2016. A chemical susceptibility profile of the *Plasmodium falciparum* transmission stages by complementary cell-based gametocyte assays. J Antimicrob Chemother 71:1148–1158. doi:10.1093/jac/dkv493. 26888912

[13] Robert V, Read AF, Essong J, Tchuinkam T, Mulder B, Verhave JP, Carnevale P. 1996. Effect of gametocyte sex ratio on infectivity of *Plasmodium falciparum* to *Anopheles gambiae*. Trans R Soc Trop Med Hyg 90:621–624. doi:10.1016/s0035-9203(96)90408-3. 9015496

[14] Baker DA. 2010. Malaria gametocytogenesis. Mol Biochem Parasitol 172:57–65. doi:10.1016/j.molbiopara.2010.03.019. 20381542 PMC2880792

[15] Tadesse FG, Meerstein-Kessel L, Gonçalves BP, Drakeley C, Ranford-Cartwright L, Bousema T. 2019. Gametocyte sex ratio: the key to understanding *Plasmodium falciparum* transmission? Trends Parasitol 35:226–238. doi:10.1016/j.pt.2018.12.001. 30594415 PMC6396025

[16] Delves MJ, Ruecker A, Straschil U, Lelièvre J, Marques S, López-Barragán MJ, Herreros E, Sinden RE. 2013. Male and female *Plasmodium falciparum* mature gametocytes show different responses to antimalarial drugs. Antimicrob Agents Chemother 57:3268–3274. doi:10.1128/AAC.00325-13. 23629698 PMC3697345

[17] Miguel-Blanco C, Lelièvre J, Delves MJ, Bardera AI, Presa JL, López-Barragán MJ, Ruecker A, Marques S, Sinden RE, Herreros E. 2015. Imaging-based high-throughput screening assay to identify new molecules with transmission-blocking potential against *Plasmodium falciparum* female gamete formation. Antimicrob Agents Chemother 59:3298–3305. doi:10.1128/AAC.04684-14. 25801574 PMC4432159

[18] Ruecker A, Mathias DK, Straschil U, Churcher TS, Dinglasan RR, Leroy D, Sinden RE, Delves MJ. 2014. A male and female gametocyte functional viability assay to identify biologically relevant malaria transmission-blocking drugs. Antimicrob Agents Chemother 58:7292–7302. doi:10.1128/AAC.03666-14. 25267664 PMC4249523

[19] Tao D, Ubaida-Mohien C, Mathias DK, King JG, Pastrana-Mena R, Tripathi A, Goldowitz I, Graham DR, Moss E, Marti M, Dinglasan RR. 2014. Sex-partitioning of the *Plasmodium falciparum* stage V gametocyte proteome provides insight into *falciparum*-specific cell biology. Mol Cell Proteomics 13:2705–2724. doi:10.1074/mcp.M114.040956. 25056935 PMC4188997

[20] Lasonder E, Rijpma SR, van Schaijk BCL, Hoeijmakers WAM, Kensche PR, Gresnigt MS, Italiaander A, Vos MW, Woestenenk R, Bousema T, Mair GR, Khan SM, Janse CJ, Bártfai R, Sauerwein RW. 2016. Integrated transcriptomic and proteomic analyses of *P falciparum* gametocytes: molecular insight into sex-specific processes and translational repression. Nucleic Acids Res 44:6087–6101. doi:10.1093/nar/gkw536. 27298255 PMC5291273

[21] Walzer KA, Kubicki DM, Tang X, Chi J-TA. 2018. Single-cell analysis reveals distinct gene expression and heterogeneity in male and female *Plasmodium falciparum* gametocytes. mSphere 3:E5. doi:10.1128/mSphere.00130-18. PMC5909122 29643077

[22] Miao J, Chen Z, Wang Z, Shrestha S, Li X, Li R, Cui L. 2017. Sex-specific biology of the human malaria parasite revealed from the proteomes of mature male and female gametocytes. Mol Cell Proteomics 16:537–551. doi:10.1074/mcp.M116.061804. 28126901 PMC5383777

[23] Rawlings DJ, Fujioka H, Fried M, Keister DB, Aikawa M, Kaslow DC. 1992. Alpha-tubulin II is a male-specific protein in *Plasmodium falciparum*. Mol Biochem Parasitol 56:239–250. doi:10.1016/0166-6851(92)90173-h. 1484548

[24] Schwank S, Sutherland CJ, Drakeley CJ. 2010. Promiscuous expression of α-tubulin II in maturing male and female *Plasmodium falciparum* gametocytes. PLoS One 5:e14470. doi:10.1371/journal.pone.0014470. 21209927 PMC3012678

[25] Tran PN, Brown SHJ, Mitchell TW, Matuschewski K, McMillan PJ, Kirk K, Dixon MWA, Maier AG. 2014. A female gametocyte-specific ABC transporter plays a role in lipid metabolism in the malaria parasite. Nat Commun 5:4773. doi:10.1038/ncomms5773. 25198203

[26] Gupta SK, Schulman S, Vanderberg JP. 1985. Stage-dependent toxicity of *N*-acetyl-glucosamine to *Plasmodium falciparum*. J Protozool 32:91–95. doi:10.1111/j.1550-7408.1985.tb03020.x. 3886901

[27] de Koning-Ward TF, Olivieri A, Bertuccini L, Hood A, Silvestrini F, Charvalias K, Berzosa Díaz P, Camarda G, McElwain TF, Papenfuss T, Healer J, Baldassarri L, Crabb BS, Alano P, Ranford-Cartwright LC. 2008. The role of osmiophilic bodies and Pfg377 expression in female gametocyte emergence and mosquito infectivity in the human malaria parasite *Plasmodium falciparum*. Mol Microbiol 67:278–290. doi:10.1111/j.1365-2958.2007.06039.x. 18086189

[28] López-Barragán MJ, Lemieux J, Quiñones M, Williamson KC, Molina-Cruz A, Cui K, Barillas-Mury C, Zhao K, Su X-Z. 2011. Directional gene expression and antisense transcripts in sexual and asexual stages of *Plasmodium falciparum*. BMC Genomics 12:587. doi:10.1186/1471-2164-12-587. 22129310 PMC3266614

[29] Chevalley S, Coste A, Lopez A, Pipy B, Valentin A. 2010. Flow cytometry for the evaluation of anti-plasmodial activity of drugs on *Plasmodium falciparum* gametocytes. Malar J 9:49. doi:10.1186/1475-2875-9-49. 20149239 PMC2830217

[30] Reid AJ, Talman AM, Bennett HM, Gomes AR, Sanders MJ, Illingworth CJR, Billker O, Berriman M, Lawniczak MK. 2018. Single-cell RNA-seq reveals hidden transcriptional variation in malaria parasites. Elife 7:1600. doi:10.7554/eLife.33105. PMC5871331 29580379

[31] MacRae JI, Dixon MW, Dearnley MK, Chua HH, Chambers JM, Kenny S, Bottova I, Tilley L, McConville MJ. 2013. Mitochondrial metabolism of sexual and asexual blood stages of the malaria parasite *Plasmodium falciparum*. BMC Biol 11:67. doi:10.1186/1741-7007-11-67. 23763941 PMC3704724

[32] Lamour SD, Straschil U, Saric J, Delves MJ. 2014. Changes in metabolic phenotypes of *Plasmodium falciparum in vitro* cultures during gametocyte development. Malar J 13:468. doi:10.1186/1475-2875-13-468. 25439984 PMC4289216

[33] Gulati S, Ekland EH, Ruggles KV, Chan RB, Jayabalasingham B, Zhou B, Mantel P-Y, Lee MCS, Spottiswoode N, Coburn-Flynn O, Hjelmqvist D, Worgall TS, Marti M, Di Paolo G, Fidock DA. 2015. Profiling the essential nature of lipid metabolism in asexual blood and gametocyte stages of *Plasmodium falciparum*. Cell Host Microbe 18:371–381. doi:10.1016/j.chom.2015.08.003. 26355219 PMC4567697

[34] Tran PN, Brown SHJ, Rug M, Ridgway MC, Mitchell TW, Maier AG. 2016. Changes in lipid composition during sexual development of the malaria parasite *Plasmodium falciparum*. Malar J 15:73. doi:10.1186/s12936-016-1130-z. 26852399 PMC4744411

[35] Schneider P, Reece SE, van Schaijk BCL, Bousema T, Lanke KHW, Meaden CSJ, Gadalla A, Ranford-Cartwright LC, Babiker HA. 2015. Quantification of female and male *Plasmodium falciparum* gametocytes by reverse transcriptase quantitative PCR. Mol Biochem Parasitol 199:29–33. doi:10.1016/j.molbiopara.2015.03.006. 25827756

[36] Stone W, Sawa P, Lanke K, Rijpma S, Oriango R, Nyaurah M, Osodo P, Osoti V, Mahamar A, Diawara H, Woestenenk R, Graumans W, van de Vegte-Bolmer M, Bradley J, Chen I, Brown J, Siciliano G, Alano P, Gosling R, Dicko A, Drakeley C, Bousema T. 2017. A molecular assay to quantify male and female *Plasmodium falciparum* gametocytes: results from 2 randomized controlled trials using primaquine for gametocyte clearance. J Infect Dis 216:457–467. doi:10.1093/infdis/jix237. 28931236 PMC5853855

[37] Santolamazza F, Avellino P, Siciliano G, Yao FA, Lombardo F, Ouédraogo JB, Modiano D, Alano P, Mangano VD. 2017. Detection of *Plasmodium falciparum* male and female gametocytes and determination of parasite sex ratio in human endemic populations by novel, cheap and robust RTqPCR assays. Malar J 16:468. doi:10.1186/s12936-017-2118-z. 29149898 PMC5693539

[38] Meerstein-Kessel L, Andolina C, Carrio E, Mahamar A, Sawa P, Diawara H, van de Vegte-Bolmer M, Stone W, Collins KA, Schneider P, Dicko A, Drakeley C, Felger I, Voss T, Lanke K, Bousema T. 2018. A multiplex assay for the sensitive detection and quantification of male and female *Plasmodium falciparum* gametocytes. Malar J 17:441. doi:10.1186/s12936-018-2584-y. 30497508 PMC6267050

[39] Roth JM, Sawa P, Omweri G, Osoti V, Makio N, Bradley J, Bousema T, Schallig HDFH, Mens PF. 2018. *Plasmodium falciparum* gametocyte dynamics after pyronaridine-artesunate or artemether-lumefantrine treatment. Malar J 17:223. doi:10.1186/s12936-018-2373-7. 29866116 PMC5987563

[40] Graumans W, Andolina C, Awandu SS, Grignard L, Lanke K, Bousema T. 2019. *Plasmodium falciparum* gametocyte enrichment in peripheral blood samples by magnetic fractionation: gametocyte yields and possibilities to reuse columns. Am J Trop Med Hyg 100:572–577. doi:10.4269/ajtmh.18-0773. 30608048 PMC6402936

[41] Alkema M, Reuling IJ, de Jong GM, Lanke K, Coffeng Le, van Gemert G-J, van de Vegte-Bolmer M, de Mast Q, van Crevel R, Ivinson K, Ockenhouse CF, McCarthy JS, Sauerwein R, Collins KA, Bousema T. 2020. A randomized clinical trial to compare *Plasmodium falciparum* gametocytaemia and infectivity following blood-stage or mosquito bite induced controlled malaria infection. J Infect Dis doi:10.1093/infdis/jiaa157. PMC8514191 32239171

[42] Conway DJ, Roper C, Oduola AM, Arnot DE, Kremsner PG, Grobusch MP, Curtis CF, Greenwood BM. 1999. High recombination rate in natural populations of *Plasmodium falciparum*. Proc Natl Acad Sci U S A 96:4506–4511. doi:10.1073/pnas.96.8.4506. 10200292 PMC16362

[43] Anderson TJ, Haubold B, Williams JT, Estrada-Franco JG, Richardson L, Mollinedo R, Bockarie M, Mokili J, Mharakurwa S, French N, Whitworth J, Velez ID, Brockman AH, Nosten F, Ferreira MU, Day KP. 2000. Microsatellite markers reveal a spectrum of population structures in the malaria parasite *Plasmodium falciparum*. Mol Biol Evol 17:1467–1482. doi:10.1093/oxfordjournals.molbev.a026247. 11018154

[44] Maier AG, Rug M. 2013. *In vitro* culturing *Plasmodium falciparum* erythrocytic stages. Methods Mol Biol 923:3–15. doi:10.1007/978-1-62703-026-7_1. 22990767

[45] Fivelman QL, McRobert L, Sharp S, Taylor CJ, Saeed M, Swales CA, Sutherland CJ, Baker DA. 2007. Improved synchronous production of *Plasmodium falciparum* gametocytes *in vitro*. Mol Biochem Parasitol 154:119–123. doi:10.1016/j.molbiopara.2007.04.008. 17521751

[46] Lambros C, Vanderberg JP. 1979. Synchronization of *Plasmodium falciparum* erythrocytic stages in culture. J Parasitol 65:418–420. doi:10.2307/3280287. 383936

[47] Christophers SR, Fulton JD. 1939. Experiments with isolated malaria parasites (*Plasmodium knowlesi*) free from red cells. Ann Trop Med Parasitol 33:161–170. doi:10.1080/00034983.1939.11685064.

[48] Eksi S, Williamson KC. 2002. Male-specific expression of the paralog of malaria transmission-blocking target antigen Pfs230, PfB0400w. Mol Biochem Parasitol 122:127–130. doi:10.1016/s0166-6851(02)00091-9. 12106866

[49] Pfaffl MW. 2001. A new mathematical model for relative quantification in real-time RT-PCR. Nucleic Acids Res 29:e45. doi:10.1093/nar/29.9.e45. 11328886 PMC55695

