# Supplementary figures and images for: Novel Method for the Separation of Male and Female Gametocytes of the Malaria Parasite Plasmodium falciparum That Enables Biological and Drug Discovery
Source: mSphere. 2020 Aug 12;5(4):e00671-20. doi: 10.1128/mSphere.00671-20 (PMC7426174; doi:10.1128/mSphere.00671-20)

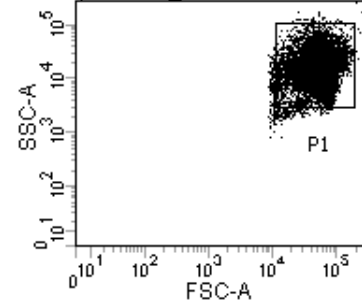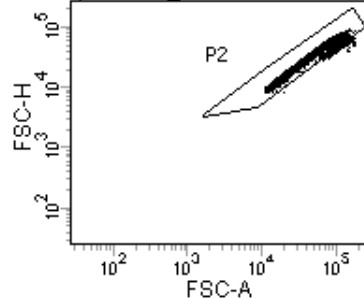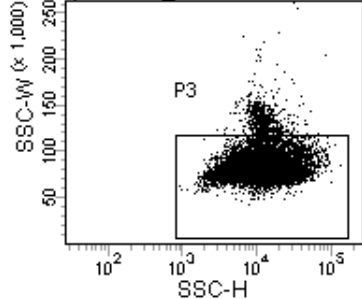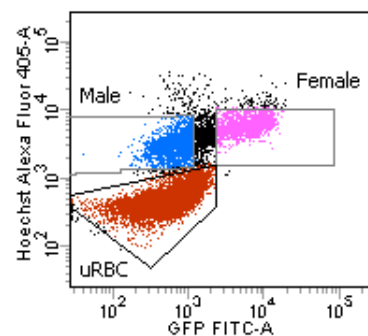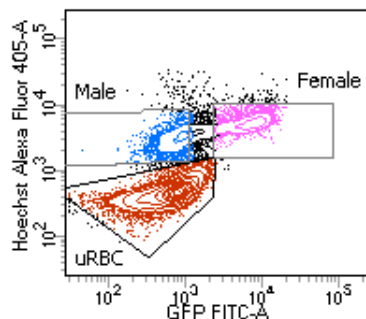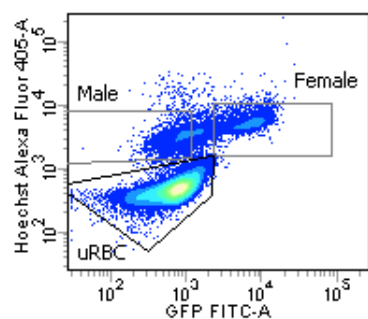

Tube: SAMPLE

| Population | #Events | %Parent | %Total |
|------------|---------|---------|--------|
| All Events | 30,000  | ####    | 100.0  |
| P1         | 29,608  | 98.7    | 98.7   |
| P2         | 29,218  | 98.7    | 97.4   |
| P3         | 28,890  | 98.9    | 96.3   |
| Female     | 1,827   | 6.3     | 6.1    |
| Male       | 1,708   | 5.9     | 5.7    |
| uRBC       | 24,225  | 83.9    | 80.8   |

Supplement: FIG S1 [file mSphere.00671-20-sf001.pdf]
