# Supplementary material for: Novel Method for the Separation of Male and Female Gametocytes of the Malaria Parasite Plasmodium falciparum That Enables Biological and Drug Discovery
Source: mSphere. 2020 Aug 12;5(4):e00671-20. doi: 10.1128/mSphere.00671-20 (PMC7426174; doi:10.1128/mSphere.00671-20)
Supplement: TABLE S2 [file mSphere.00671-20-st002.docx]

| **Gene ID** | **Annotation** | **Name** | **López-Barragán *et al*., 2011 (day 8) gametocytes stage V** | **Lasonder et al., 2016** | | **Ratio**  **(Lasonder *et al*., 2016)** |
| --- | --- | --- | --- | --- | --- | --- |
|  |  |  |  | male | female |  |
| PF3D7_1477700 | PMM1 |  | 100.31 | 984.5 | 6.8 | 144.8 (♂︎/♀︎) |
| PF3D7_1438800 | PMM2 |  | 325.17 | 678.9 | 78.5 | 8.6  (♂︎/♀︎) |
| PF3D7_0208900 | MMA | P230p | 5.15 | 88 | 2 | 44  (♂︎/♀︎) |
| PF3D7_1426500 | FMA | gABCG2 | 152.84 | 5.6 | 247.4 | 44.17 (♀︎/♂︎) |
| PF3D7_1447600 | PFM |  | 687.62 | 45 | 2571 | 57.13 (♀︎/♂︎) |
| PF3D7_1031000 | FMB | p25 | 23350 | 822.6 | 28306.3 | 34.41 (♀︎/♂︎) |
