# Supplementary material for: Novel Method for the Separation of Male and Female Gametocytes of the Malaria Parasite Plasmodium falciparum That Enables Biological and Drug Discovery
Source: mSphere. 2020 Aug 12;5(4):e00671-20. doi: 10.1128/mSphere.00671-20 (PMC7426174; doi:10.1128/mSphere.00671-20)
Supplement: TABLE S1 [file mSphere.00671-20-st001.docx]

| Stage | Gene Target | Primers (forward/reverse) |
| --- | --- | --- |
| Female | PF3D7_1426500  FMA (gABCG2) | TGCTACCTTGCATATTTCCATTTC  TTTAAGGCTTGGGACCATCTT |
|  | PF3D7_1031000  FMB (P25) | CCATGTGGAGATTTTTCCAAATGTA  CATTTACCGTTACCACAAGTTACATTC |
|  | PF3D7_1447600  PFM | CGAAACAGGATGTGGATGGATAG  GCCACAACCACAGGTATCAA |
| Male | PF3D7_0208900  MMA (6-cys protein) | CCCAACTAATCGAAGGGATGAA  AGTACGTTTAGGAGCATTTTTTGGTAA |
|  | PF3D7_1477700  PMM1 | GAGAGGAATTAAATGCTGTTCTTAGAG  ATCAACCATCCCATCCCTATTG |
|  | PF3D7_1438800  PMM2 | CAATCCAATGATAATAACCACAAAGGAG  TCCTTCCTAATTTCATTGTCCTGAA |
| Reference | PF3D7_0317300 | AATAGTCGAAGCGGGAAGTG  CGAATTGGATTCTCCCAAATAACC |
